# Antimicrobial resistance, Extended-Spectrum β-Lactamase production and virulence genes in *Salmonella enterica* and *Escherichia coli *isolates from estuarine environment

**DOI:** 10.1371/journal.pone.0283359

**Published:** 2023-04-28

**Authors:** Saharuetai Jeamsripong, Mullika Kuldee, Varangkana Thaotumpitak, Rungtip Chuanchuen

**Affiliations:** Research Unit in Microbial Food Safety and Antimicrobial Resistance, Department of Veterinary Public Health, Faculty of Veterinary Science, Chulalongkorn University, Bangkok, Thailand; Nitte University, INDIA

## Abstract

The impact of antimicrobial resistance (AMR) on global public health has been widely documented. AMR in the environment poses a serious threat to both human and animal health but is frequently overlooked. This study aimed to characterize the association between phenotype and genotype of AMR, virulence genes and Extended-Spectrum β-Lactamase (ESBL) production from estuarine environment. The *Salmonella* (*n* = 126) and *E*. *coli* (*n* = 409) were isolated from oysters and estuarine water in Thailand. The isolates of *Salmonella* (96.9%) and *E*. *coli* (91.4%) showed resistance to at least one antimicrobial agent. Multidrug resistance (MDR) was 40.1% of *Salmonella* and 23.0% of *E*. *coli*. Resistance to sulfamethoxazole was most common in *Salmonella* (95.2%) and *E*. *coli* (77.8%). The common resistance genes found in *Salmonella* were *sul3* (14.3%), followed by *bla*_TEM_ (11.9%), and *cmlA* (11.9%), while most *E*. *coli* were *bla*_TEM_ (31.5%) and *tetA* (25.4%). The ESBL production was detected in *Salmonella* (1.6%, *n* = 2) of which one isolate was positive to *bla*_TEM-1_. Eight *E*. *coli* isolates (2.0%) were ESBL producers, of which three isolates carried *bla*_CTX-M-55_ and one isolate was *bla*_TEM-1_. Predominant virulence genes identified in *Salmonella* were *invA* (77.0%), *stn* (77.0%), and *fimA* (69.0%), while those in *E*. *coli* isolates were *stx1* (17.8%), *lt* (11.7%), and *stx2* (1.2%). Logistic regression models showed the statistical association between resistance phenotype, virulence genes and ESBL production (*p* < 0.05). The findings highlighted that estuarine environment were potential hotspots of resistance. One Health should be implemented to prevent AMR bacteria spreading.

## Introduction

Antimicrobial resistance (AMR) has been recognized as one of the greatest challenges endangering the health of people, animals, and the environment. One Health approach has been applied for managing and controlling AMR at national and international levels. The Unites States Center for Disease Control and Prevention (U.S. CDC) estimated that greater than 2.8 million people and at least 35,000 deaths are affected by AMR in the U.S. annually [1]. Without a global response to AMR, it has been predicted that AMR could cause 10 million deaths annually by 2050 [2]. The global action plan to tackle AMR lists strengthening knowledge through AMR surveillance as one of the important measures to address the global AMR issue [3]. However, AMR monitoring and surveillance in the environment is rather limited and not harmonized due to several pathways responsible for AMR releasing to the environment. Therefore, knowing the magnitude of the AMR in the environment is needed for estimating impact on human and animal health.

*Salmonella* spp. is one of the most frequently isolated foodborne pathogens and a major public health threat worldwide. Humans usually get infected with *Salmonella* by consumption of contaminated food and water. Different food commodities, including poultry, swine, fish, shellfish, and produce were linked to salmonellosis in humans [4]. Salmonellosis causes 93.8 human cases and almost 155,000 deaths annually [5]. *Salmonella* contains many virulence factors that play a crucial role in the ability to infect the host cells and propagate. *Salmonella* virulence factors enhancing pathogenesis include *Salmonella* pathogenicity islands (SPIs), *Salmonella* virulence plasmids, pili, and enterotoxin [6, 7]. Despite a particular public health concern, knowledge of abundance of genotypic diversity of resistant and virulent *Salmonella* isolated from the environment is still limited.

Bivalve mollusks serve as useful and practical bioindicators of environmental fecal contamination. They have the capacity to accumulate nutrients, chemicals, and various microorganisms [8]. *E*. *coli* has been used to monitor fecal contamination and AMR in bacteria from food-producing animals for public purposes [9, 10]. Resistant *E*. *coli* can spread and transfer their resistance determinants to inter- and intra-bacterial species. A previous metagenomic analysis in untreated sewage revealed that multidrug resistance (MDR) bacteria were commonly found in waste disposed to the environment [11]. Therefore, the estuarine environment is the area of particular concern due to the high diversity and abundance of resistant bacteria that potentially pose a significant public health threat [12].

Extended-Spectrum β-Lactamases (ESBLs) are a group of enzymes that confer resistance to penicillins, third and fourth generation cephalosporins, and monobactams [13]. The spread of various types of ESBL-producing *E*. *coli* and *Salmonella* has been reported in different sectors, including human, livestock animals, and aquaculture. The genes encoding of ESBL are associated with mobile genetic elements, which can horizontally transfer to other bacterial species [14]. Infection of ESBL-producing bacterial pathogens in patients has been increasingly reported to be associated with treatment failure and increase morbidity and mortality rates due to limited effective antibiotics. Therefore, the objectives of this study were to examine phenotypic and genotypic AMR, virulence genes, and ESBL production, and to build statistical models of the association between the most common resistance phenotype and other resistance phenotype and genotypes, virulence genes, and ESBL production among *Salmonella* and *E*. *coli* isolated from oyster and estuarine water samples.

## Materials and methods

### Bacterial strains

*Salmonella* (*n* = 126) and *E*. *coli* (*n* = 409) isolates were collected from stored collection strains in the Department of Veterinary Public Health, Faculty of Veterinary Science, Chulalongkorn University. All isolates were stored in 20% glycerol stock solution at -80°C. The *Salmonella* isolates were collected from oysters (*n* = 123) and estuarine water (*n* = 3), whereas the *E*. *coli* isolates were retrieved from oysters (*n* = 250) and estuarine water (*n* = 159) samples. Oysters and estuarine waters were collected monthly between April 2016 and March 2017 from Phang Nga province in southern Thailand as previously described [15]. The oysters were wild caught, and not exposed to antimicrobials.

### Antimicrobial susceptibility testing

The agar dilution method was performed to determine minimum inhibitory concentrations (MICs) according to the Clinical and Laboratory Standard Institute [16]. Eight antimicrobials and their breakpoints were ampicillin (32 µg/ml), chloramphenicol (32 µg/ml), ciprofloxacin (4 µg/ml), gentamicin (8 µg/ml), streptomycin (32 µg/ml), sulfamethoxazole (512 µg/ml), tetracycline (16 µg/ml) and trimethoprim (16 µg/ml). *E*. *coli* ATCC 25922, *Pseudomonas aeruginosa* ATCC 27853 and *Staphylococcus aureus* ATCC 29213 were used for quality control. The multidrug resistance (MDR) was classified as resistance to at least three groups of antimicrobials.

### Detection of AMR gene

All isolates were tested for the presence of AMR genes including genes represented to ampicillin (*bla*_TEM_), chloramphenicol (*catA*, *catB* and *cmlA*), quinolone (*qnrA*, *qnrB*, and *qnrS*), aminoglycosides (*acc(3)IV* and *aadA1)*, streptomycin (*strA* and *strB*), tetracycline (*tetA* and *tetB*), sulfamethoxazole (*sul1*, *sul2*, and *sul3*), and trimethoprim (*dfrA1* and *dfrA12*) (Table 1). Conventional PCR was performed to detect most AMR genes, except genes corresponding to quinolone and sulfamethoxazole, which were used multiplex PCR. DNA templates of all *E*. *coli* and *Salmonella* were prepared using whole cell boiling technique [17]. Toptaq PCR Master Mix Kit (Merck, Munich, Germany) were followed as manufacturer’s instruction. The PCR products were separated by gel electrophoresis using 1.5% agarose gel in 1X Tris-acetate/EDTA. Gels were stained with Redsafe™ Nucleic Acid Staining Solution (iNtRon Biotechnology, Seongnam, South Korea) and visualized PCR products under UV light using Omega Fluor™ gel documentation system.

**Table 1:** Primer used and PCR condition for antimicrobial resistance genes.

Gene	Primer	Primer sequence	Denaturation	Cycle	Temperature and time (30 cycles)			Final	Amplicon size (bp)	Reference
					Denaturation	Annealing	Extension	Extension		
*catA*	catA-F	CCAGACCGTTCAGCTGGATA	5 min at 94°C	30	45 s at 95°C	45 s at 55°C	10 s at 72°C	10 min at 72°C	454	[18]
	catA-R	CATCAGCACCTTGTCGCCT								
*catB*	catB-F	CGGATTCAGCCTGACCACC	5 min at 94°C	30	45 s at 95°C	45 s at 55°C	10 s at 72°C	10 min at 72°C	461	[18]
	catB-R	ATACGCGGTCACCTTCCTG								
*cmlA*	cmlA-F	TGGACCGCTATCGGACCG	5 min at 94°C	30	45 s at 94°C	45 s at 57°C	1 min at 72°C	5 min at 72°C	641	[18]
	cmlA-R	CGCAAGACACTTGGGCTGC								
*qnrA*	qnrA-F	AGAGGATTTCTCACGCCAGG	10 min at 95°C	35	60 s at 95°C	60 s at 54°C	60 s at 72°C	10 min at 72°C	580	[19]
	qnrA-R	TGCCAGGCACAGATCTTGAC								
*qnrB*	qnrB-F	GGMATHGAAATTCGCCACTG	10 min at 95°C	35	60 s at 95°C	60 s at 54°C	60 s at 72°C	10 min at 72°C	264	[19]
	qnrB-R	TTTGCYGYYCGCCAGTCGAAC								
*qnrS*	qnrS-F	GCAAGTTCATTGAACAGGGT	10 min at 95°C	35	60 s at 95°C	60 s at 54°C	60 s at 72°C	10 min at 72°C	428	[19]
	qnrS-R	TCTAAACCGTCGAGTTCGGCG								
*aac(3)IV*	aac(3)IV-F	GTGTGCTGCTGGTCCACAGC	3 min at 95°C	35	30 s at 94°C	30 s at 55°C	60 s at 72°C	10 min at 72°C	627	[20]
	aac(3)IV-R	AGTTGACCCAGGGCTGTCGC								
*aadA1*	aadA1-F	CTCCGCAGTGGATGGCGG	5 min at 95°C	30	45 s at 95°C	45 s at 55°C	45 s at 72°C	10 min at 72°C	631	[18]
	aadA1-R	GATCTGCGCGCGAGGCCA								
*strA*	strA-F	TGGCAGGAGGAACAGGAGG	15 min at 95°C	35	30 s at 94°C	1.5 min at 57°C	1.5 min at 72°C	10 min at 72°C	405	[18]
	strA-R	AGGTCGATCAGACCCGTGC								
*strB*	strB-F	GGCAGCATCAGCCTTATAATTT	15 min at 95°C	35	30 s at 94°C	1.5 min at 57°C	1.5 min at 72°C	10 min at 72°C	470	[21]
	strB-R	GTGGATCCGTCATTCATTGTT								
*tetA*	tet(A)-F	GGCGGTCTTCTTCATCATGC	5 min at 95°C	30	45 s at 94°C	1 min at 63°C	1 min at 72°C	10 min at 72°C	502	[22]
	tet(A)-R	CGGCAGGCAGAGCAAGTAGA								
*tetB*	tet(B)-F	CGCCCAGTGCTGTTGTTGTC	5 min at 95°C	30	45 s at 95°C	45 s at 55°C	45 s at 72°C	10 min at 72°C	615	[18]
	tet(B)-R	CGCGTTGAGAAGCTGAGGTG								
*sul1*	sul1-F	CGGCGTGGGCTACCTGAACG	10 min at 95°C	30	60 s at 95°C	60 s at 66°C	60 s at 72°C	10 min at 72°C	433	[22]
	sul1-R	GCCGATCGCGTGAAGTTCCG								
*sul2*	sul2-F	CGGCATCGTCAACATAACCT	10 min at 95°C	30	60 s at 95°C	60 s at 66°C	60 s at 72°C	10 min at 72°C	721	[22]
	sul2-R	TGTGCGGATGAAGTCAGCTC								
*sul3*	sul3-F	TGTGCGGATGAAGTCAGCTC	10 min at 95°C	30	60 s at 95°C	60 s at 66°C	60 s at 72°C	10 min at 72°C	244	[22]
	sul3-R	GCTGCACCAATTCGCTGAACG								
*dfrA1*	dfrA1-F	GGAGTGCCAAAGGTGAACAGC	8 min at 94°C	32	60 s at 95°C	70 s at 55°C	10 min at 72°C	10 min at 72°C	367	[23]
	dfrA1-R	GAGGCGAAGTCTTGGGTAAAAAC								
*dfrA12*	dfrA12-F	TTCGCAGACTCACTGAGGG	8 min at 94°C	32	60 s at 95°C	70 s at 55°C	10 min at 72°C	10 min at 72°C	330	[18]
	dfrA12-R	CGGTTGAGACAAGCTCGAAT								

### Phenotypic and genotypic detection of ESBL production

Disk diffusion method was used to examine ESBL production followed by CLSI standard [16]. The detection of ESBL production consists of screening and confirmation tests. Ceftazidime (30 µg), cefotaxime (30 µg), and cefpodoxime (10 µg) were used for initial screening. All isolates that showed resistance to at least one of cephalosporins were further confirmed using a combination disk diffusion method using cephalosporins combination with clavulanic acid. The positive ESBL production was interpreted by determining the difference of inhibition zone between solely cephalosporin and cephalosporin combine with clavulanic acid. The positive ESBL-production isolates were identified β-lactamases genes (*bla*_TEM_, *bla*_SHV_, *bla*_CMY-2_, and *bla*_CTX-M-55_). The *bla*_TEM_ gene was examined using conventional PCR, while *bla*_SHV_, *bla*_CMY-2_, and *bla*_CTX-M-55_ were using multiplex PCR with the specific primers as described in Table 2.

**Table 2:** Primer used and PCR condition for extended-Spectrum β-Lactamase genes.

**Gene**	**Primer**	**Primer sequence**	**Denaturation**	**Cycle**	**Temperature and time**			**Final Extension**	**Amplicon size (bp)**	**Reference**
					**Denaturation**	**Annealing**	**Extension**			
*bla*_TEM_	blaTEM-F	TTAACTGGCGAACTACTTAC	3 min at 94°C	25	60 s at 94°C	60 s at 50°C	60 s at 72°C	10 min at 72°C	247	[22]
	blaTEM-R	GTCTATTTCGTTCATCCATA								
*bla*_SHV_	blaSHV-F	AGGATTGACTGCCTTTTTG	3 min at 94°C	25	60 s at 94°C	60 s at 50°C	60 s at 72°C	10 min at 72°C	393	[22]
	blaSHV-R	ATTTGCTGATTTCGCTCG								
*bla*_CMY-2_	blaCMY-2-F	GACAGCCTCTTTCTCCACA	3 min at 94°C	25	60 s at 94°C	60 s at 60°C	60 s at 72°C	10 min at 72°C	1000	[22]
	blaCMY-2-R	GGACACGAAGGCTACGTA								
*bla*_CTX-M_	blaCTX-M-F	CGATGTGCAGTACCTAA	3 min at 94°C	25	60 s at 94°C	60 s at 60°C	60 s at 72°C	10 min at 72°C	585	[24]
	blaCTX-M-R	AGTGACCAGAATCAGCGG								
*bla*_CTX-M_ group 1	blaCTX-M group1-F	TTAGGAARTGTGCCGCTGYA	10 min at 94°C	30	40 s at 94°C	40 s at 60°C	60 s at 72°C	7 min at 72°C	688	[25]
	blaCTX-M group1-R	CGATATCGTTGGTGGTRCCAT								

### Nucleotide sequence

PCR amplicons of positive ESBL production isolates were purified using GeneJET PCR purification kit (Thermo Fisher Scientific, Vilnius, Lithuania) and submitted for DNA sequencing (Bionics Co., ltd., Gyeonggi-Do, Republic of Korea). The result of the DNA sequence was blasted and aligned with references embedded in GenBank database available from the National Centre for Biotechnology Information (NCBI) (http://www.ncbi.nlm.nih.gov/ BLAST) (accession number OQ282894-OQ282896).

### Detection of virulence genes

Virulence genes of *Salmonella*, including invasin (*invA*), fimbrial protein (*fimA*), and enterotoxin (*stn*) genes were observed (Table 3). Heat-labile toxin (*lt*), heat-stable toxin (*st*), STEC (*stx1* and *stx2*) and EPEC for attaching and effacing protein (*eae*) were examined in all *E*. *coli* isolates. Most of virulence genes were detected using conventional PCR. The detection of *stx1* and *stx2* genes was performed by multiplex PCR.

**Table 3:** Primer used and PCR condition for virulence gene in *E*. *coli* and *Salmonella* isolates.

Gene	Primer	Primer sequence	Denaturation	Cycle	Temperature and time			Final Extension	Amplicon size (bp)	Reference
					Denaturation	Annealing	Extension			
*lt*	lt-F	TCTCTATGCATACGGAG	5 min at 95°C	30	60 s at 95°C	60 s at 55°C	60 s at 72°C	10 min at 72°C	322	[26]
	lt-R	CCATACTGATTGCCGCAATT								
*st*	st-F	TGCTAAACCAGTAGAGTCTTCAAAA	5 min at 95°C	30	60 s at 95°C	60 s at 55°C	60 s at 72°C	10 min at 72°C	138	[26]
	st-R	GCAGGCTTACAACACAATTCACAGCAG								
*stx1*	stx1-F	CAACACTGGATGATCTCAG	5 min at 94°C	35	60 s at 94°C	60 s at 55°C	60 s at 72°C	10 min at 72°C	349	[22]
	stx1-R	CCCCCTCAACTGCTAATA								
*stx2*	stx2-F	ATCAGTCGTCACTCACTGGT	5 min at 94°C	35	60 s at 94°C	60 s at 55°C	60 s at 72°C	10 min at 72°C	110	[22]
	stx2-R	CTGCTGTCACAGTGACAAA								
*eae*	eae-F	CCCGAATTCGGCACAAGCATAAGC	5 min at 95°C	30	60 s at 95°C	60 s at 55°C	60 s at 72°C	10 min at 72°C	881	[27]
	eae-R	CCCGGATCCGTCTCGCCAGTATTCG								
*fimA*	fimA-F	CCTTTCTCCATCGTCCTGAA	2 min at 95°C	35	30 s at 95°C	30 s at 55°C	60 s at 72°C	5 min at 72°C	85	[28]
	fimA-R	TGGTGTTATCTGCCTGACCA								
*stn*	stn-F	CTTTGGTCGTAAAATAAGGCG	2 min at 95°C	35	30 s at 95°C	30 s at 55°C	60 s at 72°C	5 min at 72°C	260	[28]
	stn-R	TGCCCAAAGCAGAGAGATTC								
*invA*	invA-F	GTGAAATTATCGCCACGTTCGGGCAA	2 min at 95°C	35	30 s at 95°C	30 s at 58°C	60 s at 72°C	5 min at 72°C	284	[28]
	invA-R	TCATCGCACCGTCAAAGGAACC								

### Statistical analyses

Descriptive statistics were performed to identify prevalence of resistance phenotype and genotype, resistance pattern, MDR, virulence genes, and ESBL production of *E*. *coli* and *Salmonella* isolates. Logistic regression analysis was used to examine the association among AMR, virulence genes and ESBL production. The dependent variable was the highest resistance rate, and independent variables included resistance genes, resistance phenotype, virulence genes, ESBL production and MDR. A *p*-value and confidence intervals of regression analyses were adjusted for potential correlated data within type of sample (oysters and estuarine waters) using robust variant estimator. Univariate analysis was performed to screen for potential significance of predictors. Forward selection and backward elimination were used to select potential candidates for multivariable analysis. Final regression models of *E*. *coli* and *Salmonella* were received based on *p* < 0.05 and likelihood ratio test. All statistical analyses were performed using Stata 14.0 (StataCorp, TX, USA). Two-sided hypothesis tests were used with 5% of significant level.

## Results

### Phenotype of AMR in *Salmonella* and *E*. *coli* isolates

The resistance rate of *Salmonella* (*n* = 126) and *E*. *coli* (*n* = 409) isolates were presented (Table 4). *Salmonella* resistant to at least one antibiotic was reported almost 70% (*n* = 125/129). The prevalence of MDR *Salmonella* was 23.0% (*n* = 29). The most prevalence of AMR in *Salmonella* isolates was sulfamethoxazole (95.2%, *n* = 120/126), followed by trimethoprim (37.3%, *n* = 47/126), and ampicillin (36.5%, *n* = 46/126). The AMR pattern found in *Salmonella* isolates were SUL (37.3%, *n* = 47/126), AMP-SUL-TET-TRI (11.1%, *n* = 14/126), and SUL-TRI (10.3%, *n* = 13/126), respectively.

**Table 4:** Resistance rate of *E*. *coli* (*n* = 409) and *Salmonella* (*n* = 126) isolates from oyster and estuarine water samples.

Antimicrobial agent	Number of AMR isolates (%)					
	*E*. *coli* isolates			*Salmonella* isolates		
	Oyster (*n* = 250)	Estuarine water (*n* = 159)	Total (*n* = 409)	Oyster (*n* = 123)	Estuarine water (*n* = 3)	Total (*n* = 126)
Ampicillin	124 (49.6%)	102 (64.2%)	226 (55.3%)	46 (37.4%)	-	46 (36.5%)
Chloramphenicol	38 (15.2%)	38 (23.9%)	76 (18.6%)	13 (10.6%)	-	13 (10.3%)
Ciprofloxacin	12 (4.8%)	8 (5.0%)	20 (4.9%)	11 (8.9%)	-	11 (8.7%)
Gentamicin	11 (4.4%)	9 (5.7%)	20 (4.9%)	12 (9.8%)	-	12 (9.5%)
Streptomycin	75 (30.0%)	57 (35.8%)	132 (32.3%)	11 (8.9%)	-	11 (8.7%)
Sulfamethoxazole	204 (81.6%)	114 (71.7%)	318 (77.8%)	117 (95.1%)	3 (100.0%)	120 (95.2%)
Tetracycline	95 (38.0%)	69 (43.4%)	164 (40.1%)	31 (25.2%)	-	31 (24.6%)
Trimethoprim	83 (33.2%)	67 (42.1%)	150 (36.7%)	47 (38.2%)	-	47 (37.3%)
Ceftazidime	-	5 (3.1%)	5 (1.2%)	4 (3.3%)	1 (33.3%)	5 (4.0%)
Cefotaxime	-	11 (6.9%)	11 (2.7%)	23 (18.7%)	1 (33.3%)	24 (19.0%)
Cefpodoxime	-	8 (5.0%)	8 (2.0%)	3 (2.4%)	-	3 (2.4%)

Of all *E*. *coli* isolates, 94.1% (*n* = 385/409) were resistant to at least one antibiotic, while the prevalence of MDR was observed at 40.1% (*n* = 164/409). Only 24 *E*. *coli* isolates (5.9%) were susceptible to all tested antibiotics. The predominant AMR prevalence were sulfamethoxazole (77.8%, *n* = 318/409), ampicillin (55.3%, *n* = 226/409), and tetracycline (40.1%, *n* = 164/409), respectively. The most common resistance patterns of *E*. *coli* (*n* = 409) were SUL (24.0%, *n* = 98/409), followed by AMP-CHO-STR-SUL-TET-TRI (6.8%, *n* = 28/409), AMP (5.9%, *n* = 24/409), and AMP-SUL (5.4%, *n* = 22/409).

### The presence of AMR genes in *Salmonella* and *E*. *coli* isolates

The *Salmonella* isolates (*n* = 126) from oyster and estuarine water were harbored *sul3* (14.3%, *n* = 18), followed by *bla*_TEM_ (11.9%, *n* = 15), *cmlA* (11.9%, *n* = 15), *tetA* (11.1%, *n* = 14), and *dfrA12* (9.5%, *n* = 12), while *catA*, *catB*, *qnrA*, *aac(3)IV*, *aadA1*, *strB*, *tetB*, *sul1*, *sul2*, and *dfrA1* were not observed (Table 5). The *bla*_TEM-1_ (31.5%, *n* = 129), *tetA* (25.4%, *n* = 104), and *strA* (14.9%, *n* = 61) were predominant resistance genes in the *E*. *coli* isolates, while *qnrA*, *aac(3)IV*, and *strB* were absent (Table 5).

**Table 5:** Genotypic characterization of *E*. *coli* (*n* = 409) and *Salmonella* (*n* = 126) isolated from oysters and estuarine waters.

Gene	Number of AMR isolates (%)					
	*E*. *coli* isolates			*Salmonella* isolates		
	Oyster (*n* = 250)	Estuarine water (*n* = 159)	Total (*n* = 409)	Oyster (*n* = 123)	Estuarine water (*n* = 3)	Total (*n* = 126)
*catA*	4 (1.6%)	3 (1.9%)	7 (1.7%)	-	-	-
*catB*	1 (0.4%)	1 (0.6%)	2 (0.5%)	-	-	-
*cmlA*	18 (7.2%)	20 (12.6%)	38 (9.3%)	15 (12.2%)	-	15 (11.9%)
*qnrA*	-	-	-	-	-	-
*qnrB*	3 (1.2%)	2 (1.3%)	5 (1.2%)	4 (3.3%)	-	4 (3.2%)
*qnrS*	25 (10.0%)	24 (15.1%)	49 (12.0%)	10 (8.1%)	-	10 (7.9%)
*aac(3)IV*	-	-	-	-	-	-
*aadA1*	26 (10.4%)	21(13.2%)	47 (11.5%)	-	-	-
*strA*	28 (11.2%)	33 (20.8%)	61 (14.9%)	5 (4.1%)	-	5 (4.0%)
*strB*	-	-	-	-	-	-
*tetA*	54 (21.6%)	50 (31.4%)	104 (25.4%)	14 (11.4%)	-	14 (11.1%)
*tetB*	10 (4.0%)	6 (3.8%)	16 (3.9%)	-	-	-
*sul1*	4 (1.6%)	1 (0.6%)	5 (1.2%)	-	-	-
*sul2*	19 (7.6%)	32 (20.1%)	51 (12.5%)	-	-	-
*sul3*	19 (7.6%)	22 (13.8%)	41 (10.0%)	18 (14.6%)	-	18 (14.3%)
*dfrA1*	5 (2.0%)	3 (1.9%)	8 (2.0%)	-	-	-
*dfrA12*	17 (6.8%)	18 (11.3%)	35 (8.6%)	12 (9.8%)	-	12 (9.5%)
*bla*_TEM-1_	69 (27.6%)	60 (37.7%)	129 (31.5%)	15 (12.2%)	-	15 (11.9%)

### ESBL production in *Salmonella* and *E*. *coli* isolate

For all *Salmonella* isolates, two (1.6%) isolates from oyster samples (serovars Augustenborg and II) (data not shown) were confirmed as ESBL producing isolates. The serovar II of ESBL-producing *Salmonella* was *bla*_TEM-1_ positive. Eight (2.0%) out of 409 *E*. *coli* isolates from estuarine water were ESBL-producers, of which three isolates were positive for *bla*_CTX-M-55_. None of ESBL-producing *E*. *coli* isolates was retrieved from oyster samples.

### Distribution of virulence genes

Out of 409 *E*. *coli* isolates, 50.0% of *lt*, 41.1% of *stx1* and 40% of *stx2* were MDR. Of all *Salmonella* isolates, *invA* (77.0%, *n* = 97/126), *stn* (77.0%, *n* = 97/126), and *fimA* (69.0%, *n* = 87/126) were the frequently found virulent genes (Table 6). Among these, the *Salmonella* isolates positive to *invA* (28.9%), *fimA* (29.9%), and *stn* (26.8%) were MDR. The *E*. *coli* isolates mainly harbored *stx1* (17.8%, *n* = 73/409), followed by *lt* (11.7%, *n* = 48/409) and *stx2* (1.2%, *n* = 5/409) (Table 6). The *E*. *coli* isolates from oyster samples predominantly contained *stx1* (10.3%) and *lt* (9.5%), while the isolates from estuarine water commonly carried *stx1* (7.6%). The *st* and *stx2* genes were not detected in *E*. *coli* isolated from estuarine water.

**Table 6:** Virulence genes of *E*. *coli* (*n* = 409) and *Salmonella* (*n* = 126) isolated from oysters and estuarine waters.

Virulent gene	Number of positive isolates (%)		
	Oyster	Estuarine water	Total
***E*. *coli***			
*lt*	39 (9.5%)	9 (2.2%)	48 (11.7%)
*st*	1 (0.2%)	-	1 (0.2%)
*stx1*	42 (10.3%)	31 (7.6%)	73 (17.8%)
*stx2*	5 (1.2%)	-	5 (1.2%)
*eae*	-	1 (0.2%)	1 (0.2%)
***Salmonella***			
*invA*	94 (74.6%)	3 (2.4%)	97 (77.0%)
*fimA*	84 (66.7%)	3 (2.4%)	87 (69.0%)
*stn*	94 (74.6%)	3 (2.4%)	97 (77.0%)

### Co-existence among AMR, ESBL production, and virulence genes

One *Salmonella* isolated from oyster sample harbored *bla*_TEM-1_ with MDR to ampicillin, chloramphenicol, sulfamethoxazole, trimethoprim, and tetracycline. The latter was also positive to *invA*, *sul3*, *cmlA*, and *dfrA12* genes. An ESBL-producing *E*. *coli* isolated from cultivation water harbored *bla*_TEM-1_ was resistant to ampicillin, while three ESBL-producing *E*. *coli* with *bla*_CTX-M-55_ were MDR.

### Association between AMR, virulence genes, ESBL production, and MDR

Based on logistic regression analyses, the sulfamethoxazole-resistant *Salmonella* were statistically associated with the presence of ampicillin resistance (OR = 3.06), trimethoprim resistance (OR = 1.47), and *invA* (OR = 1.95) (*p* < 0.0001) compared with those isolates that were not resistant to sulfamethoxazole. The sulfamethoxazole-resistant *Salmonella* were negatively associated with ESBL production (*p* < 0.0001, OR = 0.02) and *stn* (*p* < 0.0001, OR = 0.56).

The *E*. *coli* isolates resistant to sulfamethoxazole were positively associated with the presence of trimethoprim (*p* = 0.027, OR = 1.55), ESBL production (*p* < 0.0001, OR = 1.83), MDR (*p* = 0.008, OR = 10.33), *addA1* (*p* = 0.002, OR = 3.05), *strA* (*p* = 0.044, OR = 2.66), and *sul3* (*p* < 0.0001, OR = 8.38) than other isolates that were susceptible to sulfamethoxazole (Table 7). However, the *E*. *coli* isolates resistant to sulfamethoxazole were negatively associated with two virulence genes, including *lt* (*p* = 0.025, OR = 0.43) and *stx* (*p* = 0.022, OR = 0.41), and *dfrA12* (*p* < 0.0001, OR = 0.10).

**Table 7:** Logistic regression model of the association between *E*. *coli* resistance to sulfamethoxazole and resistance phenotype, resistance gene, and virulence genes (*n* = 409) classified by type of samples.

Predictor	Odds ratio	Std. Err.^*a*^	95% C.I.^*b*^	*p-*value
Ampicillin	0.20	0.20	0.16–0.25	< 0.0001
Gentamicin	0.16	0.14	0.03–0.94	0.043
Trimethoprim	1.55	0.31	1.05–2.30	0.027
Cefotaxime	0.33	0.18	0.11–0.97	0.044
ESBL production	1.83	0.16	1.55–2.17	< 0.0001
MDR	10.33	9.08	1.84–57.87	0.008
*lt*	0.43	0.16	0.21–0.90	0.025
*stx*	0.41	0.16	0.19–0.88	0.022
*addA1*	3.05	1.10	1.51–6.18	0.002
*strA*	2.66	1.29	1.03–6.89	0.044
*sul3*	8.38	5.30	2.43–28.91	< 0.0001
*dfrA12*	0.10	0.05	0.040–0.27	< 0.0001
Constant	4.34	2.18	1.63–11.61	0.003

AIC^*c*^ = 344.67

^*a*^Std. Err. is Standard Error

^*b*^C.I.: Confidence Interval

^*c*^AIC: Akaike Information Criteria

## Discussion

One of the main findings of this study is more than 90% of *Salmonella* and *E*. *coli* from fresh oyster (96.7%; *n* = 119/123 of *Salmonella* and 95.2%; *n* = 238/250 of *E*. *coli* isolates) and estuarine water (100.0%; *n* = 3/3 of *Salmonella* and 92.5%; *n* = 147/159 of *E*. *coli* isolates) samples were resistant to at least one antimicrobial. MDR *Salmonella* (23.0%) and *E*. *coli* (40.0%) were also isolated, even though the oysters were received from wild caught with no evidence of antimicrobial use. This cultivation site could be contaminated from nearby communities and agriculture according to previous studies [29, 30], so that trackback investigation to identify the source of AMR in estuarine environment is recommended. Estuarine water was considered a potential hotspot to surveillance of AMR distribution in the environment [30]. Humans can be infected with AMR bacteria by eating aquatic animals or direct contact with contaminated environment. Resistant bacteria found in this study are considered as important estuarine environmental pollutants that can adversely affect food security and public health.

In this study, the highest resistance rates acquired by both *Salmonella* (95.2%) and *E*. *coli* (77.8%) was to sulfamethoxazole. However, a previous study reported the lower prevalence of *Salmonella* resistant to sulfonamides (56.5%) in retail aquaculture products such as shellfish, calm, fish, shrimp, and others in Shanghai [31]. The high prevalence of sulfamethoxazole observed in this study may be widely used in human and animal medicine because this antimicrobial can be used to treat and prevent many bacterial infections at affordable cost [32].Therefore, it is possible that sulfamethoxazole could disseminate and accumulate to the environment. Sulfamethoxazole is effective against both Gram-negative and Gram-positive bacteria, including *E*. *coli* and *Listeria monocytogenes*. This antimicrobial agent is commonly used to treat urinary tract infection, bronchitis, and prostatitis. In veterinary medicine, sulfonamides have been used in swine and cattle production for treatment of urinary and respiratory tract infection. High concentration of sulfonamides in the environment has been indicated in livestock manure due to the common use of this antimicrobial [33, 34]. Sulfamethoxazole-resistant bacteria was also found in surface water and soil causing environmental pollutants as a result of the widely used in treatment of animals and humans [35]. The impact of sulfonamides contamination in the environment could result in hazardous to human health (e.g., difficult to treat of resistant bacteria, prolong hospital stay etc.), and alter microbial community [33]. However, the consequences of sulfonamide contamination in the ecosystem were still unclear [36]. Therefore, the removal of these resistant bacteria from healthcare facilities, livestock farms, and communities are needed to reduce the contamination to the coastal environment. Previous studies developed the removal of sulfonamides by using anaerobic membrane bioreactor in swine wastewater, and the use of *Pleurotus eryngii* for degradation of sulfonamides [37, 38].

Besides sulfamethoxazole resistance, the high resistance was observed for trimethoprim (37.3%) and ampicillin (36.5%) in *Salmonella*, and for ampicillin (55.3%), and tetracycline (40.1%) in *E*. *coli*. These findings agreed with previous studies conducted in aquatic animals and estuarine environment [39, 40]. High resistance rates to ampicillin (100%) and erythromycin (83.33%) in *Salmonella* isolates were previously reported in water and sediment [41]. The high resistance to sulfamethoxazole, ampicillin, tetracycline, and trimethoprim observed in this study was commonly reported in humans and animals [42–45]. In Thailand, the molecular epidemiology and association of AMR among of *E*. *coli* and *S*. *enterica* have been extensively investigated from pigs, pork, and humans indicating the potential risk of AMR spreading [43, 46]. Even though the precise genetic relationship information is still lacking, the observations of resistance to these antimicrobials in humans, food-producing animals, and environment in the same country confirm that AMR is a complex One Health issue.

*S*. *enterica* serovar Paratyphi B causes a serious disease, Paratyphoid, in humans. The serovars Paratyphi B poses a significant health risk due to being associated with sporadic outbreaks of human infection and multistage outbreaks of seafood products [46–48]. The symptoms of paratyphoid infection in humans are fever, loss of appetite, weakness, headaches, diarrhea, and may be a life-threatening multi-systemic illness. The pathogens were recently isolated from poultry and poultry meat from Europe and Latin America [49]. A study reported that serovar Paratyphi B was isolated from oysters (22.7%) in Thailand [15]. In this study, the serovars Paratyphi B was isolated from oysters (13.5%, *n* = 17/126), and all these isolates were resistant to at least one antimicrobial and 29.4% (*n* = 5/17) were MDR. More than 75% (*n* = 13/17) of these isolates contained virulence genes (i.e., *fimA* and *stn*), and 64.7% (*n* = 11/17) of all Paratyphi B isolates harbored *invA*. The presence of MDR Paratyphi B isolates in oysters may pose a serious threat to public health in the near future due to the difficulty in controlling strategic action.

Most resistance genes detected in this study corresponded well to observed resistance phenotype, suggesting that resistance genes were usually expressed when present. In *Salmonella* isolates, the most detected resistance genes were *sul3* (14.3%), *bla*_TEM_ (11.9%), *cmlA* (11.9%), and *tetA* (11.1%), while those in *E*. *coli* isolates were *bla*_TEM_ (31.5%), followed by *tetA* (25.4%) and *strA* (14.9%). High prevalence (91.3%) of *bla*_TEM_ gene was previously reported in oysters [50], which agreed with this study. This study observed the presence of β-lactamase encoding *bla*_TEM-1_ indicating a narrow spectrum activity against β-lactamase of *E*. *coli* and *Salmonella*. This indicated that the estuarine environment serves as a potential hotspot of AMR bacteria carrying resistance determinants that may be transferred to bacterial pathogens in humans and animals.

In this study, the occurrence of ESBL-producing *E*. *coli* (2.0%) and *Salmonella* (1.6%) was lower than in a previous study, which greatly varied in humans (11–72%), animals (0–72%), and wastewater (7–79%) in West and Central Africa [51]. Greater than 40% of wastewater from Tunisia were positive to ESBL-producing *Enterobacteriaceae* [52]. In this study, *bla*_TEM-1_ (*n* = 2) and *bla*_CTX-M-55_ (*n* = 3) were reported with MDR, which agreed with previous studies in aquatic environment and migratory birds [53, 54]. Furthermore, the *bla*_TEM_ and *bla*_CTX-M_ isolates were the common widespread genes from wild fish and aquatic environment [54, 55]. More specifically, the *bla*_TEM-1_, *bla*_CTX-M-14_ and *bla*_CTX-M-15_ genes were reported from marine bivalve mollusks [8]. Even though the low rates of ESBL producing bacteria were observed in this study, the positive ESBL isolates were commonly identified MDR bacteria. Hence, the occurrence of ESBL producing bacteria that harbored MDR signifies the public health threat.

The association between resistance to sulfamethoxazole and other predictors, including AMR, MDR, virulence genes, and ESBL production were examined under the logistic regression models (Tables 7, 8). The complexity of association among resistance and virulence of *E*. *coli* and *Salmonella* was observed. Sulfamethoxazole resistance in *E*. *coli* was positively associated with trimethoprim resistance, ESBL production, MDR, and the presence of *addA1*, *strA*, and *sul3*, but these isolates were negatively associated with *lt*, *stx*, and *dfrA12*. The major concern of these findings was almost half of *E*. *coli* carrying virulence genes were MDR bacteria. A co-selection of resistance and virulence can occur through mobile genetic elements such as integrons, transposons, and integrative conjugative elements [56]. The infection of resistant and virulent pathogens is detrimental to human health since they cause difficulty to treat and increase treatment failure. On the other hand, sulfamethoxazole resistance in *Salmonella* was positively correlated with resistance to ampicillin and trimethoprim, and *invA*, but they were negatively associated with ESBL production and *stn*. This finding indicated the complexity of AMR, virulence factors and resistance determinants in the environment. A quarter of *Salmonella* carrying virulence genes were MDR. Thus, sulfamethoxazole resistance isolates can co-selection to many classes of antimicrobials, virulence genes, and ESBL production. A previous study indicated that resistance and virulence plasmids were linked simultaneously [57]. As a result, the infection of resistant and virulent bacteria may cause more complicated treatment and increase morbidity and mortality rates due to failure of bacterial treatment.

**Table 8:** Logistic regression model of the association between *Salmonella* resistance to sulfamethoxazole and resistance phenotype, resistance gene, and virulence genes (*n* = 126) classified by type of samples.

Predictor	Odds ratio	Std. Err.^*a*^	95% C.I.^*b*^	*p*-value
Ampicillin	3.06	0.20	2.70–3.47	< 0.0001
Trimethoprim	1.47	0.070	1.34–1.62	< 0.0001
ESBL production	0.02	0.0002	0.017–0.018	< 0.0001
*invA*	1.95	0.14	1.69–2.26	< 0.0001
*stn*	0.56	0.006	0.55–0.57	< 0.0001
Constant	15.43	0.41	14.65–16.26	< 0.0001

AIC^*c*^ = 44.72

^*a*^Std. Err. is Standard Error

^*b*^C.I.: Confidence Interval

^*c*^AIC: Akaike Information Criteria

Shiga toxin is bacterial exotoxin related to highly cytotoxic class II ribosome [58]. In this study, *stx1*, Shiga toxin-producing *E*. *coli* (STEC) was most frequently found in oysters and estuarine waters, while *eae* gene representing enteropathogenic *E*. *coli* (EPEC) was reported in estuarine water at a low rate (0.2%). A previous study indicated that none of virulence genes related to STEC and EPEC were identified in oysters and mussels from Atlantic Canada [29], in contrast to the results in this study. Wildlife and aquaculture, including fish and shellfish have been identified as one of important sources of STEC spillover from livestock animals [59]. The high rate of *stx1* in this study raise public health concerns of seafood safety, since major clinical signs of STEC infection in humans are bloody diarrhea, hemorrhagic colitis, and hemolytic uremic syndrome, and may be life-threatening.

The *fimA*, *stn*, and *invA* genes are common virulence genes that play an important role in the pathogenicity of *Salmonella* infection. The *fimA* gene is a common structural subunit of type 1 fimbrial protein, while *stn* is heat-labile *Salmonella* enterotoxin affecting epithelial cells [60, 61]. The *invA* gene is an important structural component of *Salmonella* pathogenicity island, which is related to invasion of gut epithelial tissues in human and animals [28]. In this study, 77.0% of *Salmonella* isolates were positive to *invA* gene, even though this gene has been used for confirmation of *Salmonella* in food animals. This agreed with previous studies where the absence of *invA* gene was found in poultry production [62, 63]. In seafood and environmental samples, some *Salmonella* isolates confirmed with biochemical test did not contain *invA* gene [64–66]. The absence of *invA* gene may be because *Salmonella* was not invasive or had other invasive mechanisms [67]. However, the absence of *invA* genes is a rare occasion. The combination of PCR and next generation sequencing (NGS) is proposed to increase sensitivity of *Salmonella* detection of resistance in environmental samples [68].

In conclusion, MDR and ESBL-producing *E*. *coli* are widespread in the estuarine environment, highlighting the need for continuing AMR monitoring programs in shellfish harvested area. Knowing the magnitude of AMR circulated in the environment can facilitate developing strategic action plans to mitigate the possible transmission of resistance bacteria among humans, animals, and environment. In addition to phenotypic detection of AMR, identification of AMR driving sources and monitoring of genetic information of resistance organisms are required to better understanding reduce the occurrence and transference of AMR in aquatic animals and estuarine waters. Oysters and estuarine water serve as overlooked natural reservoirs of AMR contamination. Awareness of seafood safety and increase personal hygiene are suggested to reduce AMR infection from seafood consumption.

## References

[1] CDC. Antibiotic resistance. Centers for Disease Control and Prevention, National Center for Emerging and Zoonotic Infectious Diseases (NCEZID), Division of Healthcare Quality Promotion (DHQP). https://www.cdc.gov/drugresistance/about.html. 2022.

[2] de Kraker ME, Stewardson AJ, Harbarth S. Will 10 million people die a year due to antimicrobial resistance by 2050?. PloS Medicine. 2016 Nov; 13:e1002184. 10.1371/journal.pmed.1002184 doi: 10.1371/journal.pmed.1002184 27898664 PMC5127510

[3] WHO. Global action plan on antimicrobial resistance. 2015. Online http://apps.who.int/iris/bitstream/10665/193736/1/9789241509763\_eng.pdf?ua = 1.

[4] EFSA and ECDC. The European Union summary report on trends and sources of zoonoses, zoonotic agents and food-borne outbreaks in 2017. European Food Safety Authority and European Centre for Disease Prevention and Control. EFSA Journal. 2018; 16:1–262. 10.2903/j.efsa.2018.5500. PMC7009540 32625785

[5] Majowicz SE, Musto J, Scallan E, Angulo FJ, Kirk M, O’Brien SJ, et al. The global burden of nontyphoidal *Salmonella* gastroenteritis. Clinical Infectious Diseases. 2010 Mar; 50(6):882–9. 10.1086/650733. 20158401

[6] Hansen-Wester I, Hensel M. *Salmonella* pathogenicity islands encoding type III secretion systems. Microbes and Infection. 2001 Jun; 3(7):549–59. 10.1016/S1286-4579(01)01411-3. 11418329

[7] Yue M, Li X, Liu D, Hu X. Serotypes, antibiotic resistance, and virulence genes of *Salmonella* in children with diarrhea. Journal of Clinical Laboratory Analysis. 2020 Dec; 34(12):e23525. 10.1002/jcla.23525. doi: 10.1002/jcla.23525 . 32797660 PMC7755775

[8] Grevskott DH, Svanevik CS, Sunde M, Wester AL, Lunestad BT. Marine bivalve mollusks as possible indicators of multidrug-resistant *Escherichia coli* and other species of the *Enterobacteriaceae* family. Frontiers in Microbiology. 2017 Jan; 8:24. 10.3389/fmicb.2017.00024. doi: 10.3389/fmicb.2017.00024 . 28149295 PMC5241299

[9] Nyirabahizi E, Tyson GH, Dessai U, Zhao S, Kabera C, Crarey E, et al. Evaluation of *Escherichia coli* as an indicator for antimicrobial resistance in Salmonella recovered from the same food or animal ceca samples. Food Control. 2020 Sep; 115:107280. 10.1016/j.foodcont.2020.107280.

[10] EFSA. European Food Safety Authority, Aerts, Battisti A, Hendriksen R, Kempf I, Teale C, Tenhagen BA, et al. Technical specifications on harmonized monitoring of antimicrobial resistance in zoonotic and indicator bacteria from food-producing animals and food. EFSA Journal. 2019; 17:e05709. https://www.efsa.europa.eu/en/efsajournal/pub/5709. 32626332 10.2903/j.efsa.2019.5709 PMC7009308

[11] Hendriksen RS, Munk P, Njage P, Van Bunnik B, McNally L, Lukjancenko O, et al. Global monitoring of antimicrobial resistance based on metagenomics analyses of urban sewage. Nature Communications. 2019 Mar 8; 10(1):1124. 10.1038/s41467-019-08853-3. doi: 10.1038/s41467-019-08853-3 . 30850636 PMC6408512

[12] Zhao Z, Zhang K, Wu N, Li W, Xu W, Zhang Y, et al. Estuarine sediments are key hotspots of intracellular and extracellular antibiotic resistance genes: A high-throughput analysis in Haihe Estuary in China. Environment International. 2020 Feb; 135:105385. 10.1016/j.envint.2019.105385. doi: 10.1016/j.envint.2019.105385 . 31855802

[13] Paterson DL, Bonomo RA. Extended-spectrum beta-lactamases: a clinical update. Clinical Microbiology Reviews. 2005 Oct; 18(4):657–86. https://journals.asm.org/doi/10.1128/CMR.18.4.657-686.2005. doi: 10.1128/CMR.18.4.657-686.2005 . 16223952 PMC1265908

[14] Ruppé É, Woerther PL, Barbier F. Mechanisms of antimicrobial resistance in Gram-negative bacilli. Annals of Intensive Care. 2015 Aug; 5:21. 10.1186/s13613-015-0061-0. doi: 10.1186/s13613-015-0061-0 . 26261001 PMC4531117

[15] Jeamsripong S, Chuanchuen R, Atwill ER. Assessment of bacterial accumulation and environmental factors in sentinel oysters and estuarine water quality from the Phang Nga estuary area in Thailand. International Journal of Environmental Research and Public Health. 2018 Sep; 15(9):1970. 10.3390/ijerph15091970. doi: 10.3390/ijerph15091970 . 30201900 PMC6165384

[16] CLSI Performance standards for antimicrobial disk and dilution susceptibility tests for bacteria isolated from animals; second informational supplement. CLSI document VET01. 2013.

[17] Dashti A, Jadaon M, Abdulsamad A, Dashti H. Heat treatment of bacteria: a simple method of DNA extraction for molecular techniques. The Journal of the Kuwait Medical Association. 2009 Jun: 41(2). https://applications.emro.who.int/imemrf/kmj_2009_41_2_117.pdf.

[18] Chuanchuen R, Padungtod P, Pathanasophon P. Antimicrobial resistance genes among *Salmonella enterica* isolates from poultry and swine in Thailand. International Journal of Infectious Diseases. 2008 Dec; 12: e117. 10.1016/j.ijid.2008.05.292.

[19] Cattoir V, Poirel L, Rotimi V, Soussy C-J and Nordmann P. Multiplex PCR for detection of plasmid-mediated quinolone resistance *qnr* genes in ESBL-producing *enterobacterial* isolates. The Journal of Antimicrobial Chemotherapy. 2007 Aug; 60(2):394–7. 10.1093/jac/dkm204. doi: 10.1093/jac/dkm204 . 17561500

[20] Stoll C, Sidhu JPS, Tiehm A, Toze S. Prevalence of clinically relevant antibiotic resistance genes in surface water samples collected from Germany and Australia. Environmental Science and Technology. 2012 Sep; 46(17):9716–26. 10.1021/es302020s. doi: 10.1021/es302020s 22846103

[21] Mala W, Kaewkes W, Tattawasart U, Wongwajana S, Faksri K, Chomvarin C. SXT element, class 1 Integron and multidrug-resistance genes of *Vibrio cholerae* isolated from clinical and environmental sources in northeast Thailand. Southeast Asian Journal of Tropical Medicine in Public Health. 2016 Sep; 47(5):957–66. https://www.thaiscience.info/journals/Article/TMPH/10983783.pdf . 29620801

[22] Khan SB, Khan MA, Ahmad I, ur Rehman T, Ullah S, Dad R, et al. Phenotypic, genotypic antimicrobial resistance and pathogenicity of *Salmonella enterica* serovars Typimurium and Enteriditis in poultry and poultry products. Microbial Pathogenesis. 2019 Apr; 129:118–24. 10.1016/j.micpath.2019.01.046. doi: 10.1016/j.micpath.2019.01.046 . 30738177

[23] Shahrani M, Safarpoor F, Momtaz H. Characterization of *Escherichia coli* virulence genes, pathotypes and antibiotic resistance properties in diarrheic calves in Iran. Biological Research. 2014 Jun; 47(1):28. 10.1186/0717-6287-47-28. doi: 10.1186/0717-6287-47-28 . 25052999 PMC4105491

[24] Batchelor M, Hopkins K, Threlfall EJ, Clifton-Hadley FA, Stallwood AD, Davies RH, et al. *bla*_CTX-M_ genes in clinical *Salmonella* isolates recovered from humans in England and Wales from 1992 to 2003. Antimicrobial Agents and Chemotherapy. 2005 Apr; 49(4): 1319–22. 10.1128/AAC.49.4.1319-1322.2005. doi: 10.1128/AAC.49.4.1319-1322.2005 . 15793104 PMC1068621

[25] Dallenne C, Da Costa A, Decre D, Favier C, Arlet G. Development of a set of multiplex PCR assays for the detection of genes encoding important ß-lactamases in *Enterobacteriaceae*. Journal of Antimicrobial Chemotherapy. 2010 Jan; 65: 490–495. https://doi.org/ 10.1093/jac/dkp498. doi: 10.1093/jac/dkp498 . 20071363

[26] Hinthong W, Pumipuntu N, Santajit S, Kulpeanprasit S, Buranasinsup S, Sookrung N, et al. Detection and drug resistance profile of *Escherichia coli* from subclinical mastitis cows and water supply in dairy farms in Saraburi Province, Thailand. Peer Journal. 2017 Jun; 5:e3431. 10.7717/peerj.3431. doi: 10.7717/peerj.3431 . 28626609 PMC5472034

[27] Toma C, Lu Y, Higa N, Nakasone N, Chinen I, Baschkier A, et al. Multiplex PCR assay for identification of human diarrheagenic *Escherichia coli*. Journal of Clinical Microbiology. 2003 Jun; 41(6): 2669–71. 10.1128/JCM.41.6.2669-2671.2003. doi: 10.1128/JCM.41.6.2669-2671.2003 . 12791900 PMC156568

[28] Kumar R, Surendran PK, Thampuran N. Detection and characterization of virulence factors in lactose positive and lactose negative *Salmonella* serovars isolated from seafood. Food Control. 2009 April; 20(4):376–80. 10.1016/j.foodcont.2008.06.005.

[29] Rees EE, Davidson J, Fairbrother JM, St Hilaire S, Saab M, McClure JT. Occurrence and antimicrobial resistance of *Escherichia coli* in oysters and mussels from Atlantic Canada. Foodborne Pathogens and Disease. 2015 Feb; 12(2):164–9. 10.1089/fpd.2014.1840. doi: 10.1089/fpd.2014.1840 . 25551332

[30] Singer AC, Shaw H, Rhodes V, Hart A. Review of antimicrobial resistance in the environment and its relevance to environmental regulators. Frontiers in Microbiology. 2016 Nov; 7:1728. 10.3389/fmicb.2016.01728. doi: 10.3389/fmicb.2016.01728 . 27847505 PMC5088501

[31] Zhang J, Yang X, Kuang D, Shi X, Xiao W, Zhang J, et al. Prevalence of antimicrobial resistance of non-typhoidal *Salmonella* serovars in retail aquaculture products. International Journal of Food Microbiology. 2015 Oct; 210:47–52. 10.1016/j.ijfoodmicro.2015.04.019. doi: 10.1016/j.ijfoodmicro.2015.04.019 . 26093990

[32] Kemnic TR, Coleman M. Trimethoprim Sulfamethoxazole. In: StatPearls. Treasure Island (FL): StatPearls Publishing; 2022 Jan; https://www.ncbi.nlm.nih.gov/books/NBK513232/. 30020604

[33] Baran W, Adamek E, Ziemiańska J, Sobczak A. Effects of the presence of sulfonamides in the environment and their influence on human health. Journal of Hazardous Materials. 2011 Nov; 196:1–15. 10.1016/j.jhazmat.2011.08.082. doi: 10.1016/j.jhazmat.2011.08.082 21955662

[34] Checcucci A, Trevisi P, Luise D, Modesto M, Blasioli S, Braschi I, et al. Exploring the animal waste resistome: The spread of antimicrobial resistance genes through the use of livestock manure. Frontiers in Microbiology. 2020 Jul; 11:1416. 10.3389/fmicb.2020.01416. doi: 10.3389/fmicb.2020.01416 . 32793126 PMC7387501

[35] Hruska K, Franek M. Sulfonamides in the environment: a review and a case report. Veterinární Medicína 2012 Jan; 57(1): 1–35 10.17221/4969-VETMED.

[36] Kergoat L, Besse-Hoggan P, Leremboure M, Beguet J, Devers M, Martin-Laurent F, et al. Environmental concentrations of sulfonamides can alter bacterial structure and induce diatom deformities in freshwater biofilm communities. Frontiers in Microbiology. 2021 May; 12:643719. 10.3389/fmicb.2021.643719. doi: 10.3389/fmicb.2021.643719 . 34025605 PMC8137839

[37] Cheng D, Ngo HH, Guo W, Chang SW, Nguyen DD, Nguyen QA, et al. Improving sulfonamide antibiotics removal from swine wastewater by supplying a new pomelo peel derived biochar in an anaerobic membrane bioreactor. Bioresource Technology. 2021 Jan; 319:124160. 10.1016/j.biortech.2020.12416010.1016/j.biortech.2020.124160. doi: 10.1016/j.biortech.2020.12416010.1016/j.biortech.2020.124160 33010716

[38] Baran W, Adamek E, Włodarczyk A, Lazur J, Opoka W, Muszyńska B. 2021. The remediation of sulfonamides from the environment by *Pleurotus eryngii* mycelium. Efficiency, products and mechanisms of mycodegradation. Chemosphere. 2021 Jan; 262:128026. 10.1016/j.chemosphere.2020.128026. 33182090

[39] Oliveira AMS, Baraúna RA, Marcon DJ, Lago LAB, Silva A, Lusio J, et al. Occurrence, antibiotic-resistance and virulence of E. coli strains isolated from mangrove oysters (*Crassostrea gasar*) farmed in estuaries of Amazonia. Marine Pollution Bulletin. 2020 Aug; 157:111302. 10.1016/j.marpolbul.2020.111302. doi: 10.1016/j.marpolbul.2020.111302 . 32658670

[40] Elbashir S, Parveen S, Schwarz J, Rippen T, Jahncke M, DePaola A. Seafood pathogens and information on antimicrobial resistance: A review. Food Microbiology. 2018 Apr; 70:85–93. 10.1016/j.fm.2017.09.011. doi: 10.1016/j.fm.2017.09.011 . 29173644

[41] Hossain MS, Khaleque HM, Mazumder F, Mahbub KR. Prevalence of multidrug resistant *Salmonella* in shrimp of Dhaka city. Microbiology Journal. 2013; 3(1):21–8. https://ascidatabase.com/ascidetail.php?doi=mj.2013.21.28&kw=.

[42] Phongaran D, Khang-Air S, Angkititrakul S. Molecular epidemiology and antimicrobial resistance of *Salmonella* isolates from broilers and pigs in Thailand. Veterinary World. 2019 Aug;12(8):1311–8. 10.14202/vetworld.2019.1311-1318. doi: 10.14202/vetworld.2019.1311-1318 . 31641313 PMC6755382

[43] Sinwat N, Angkittitrakul S, Chuanchuen R. Characterization of antimicrobial resistance in *Salmonella enterica* isolated from pork, chicken meat, and humans in Northeastern Thailand. Foodborne Pathogens and Disease. 2015 Sep; 12(9):759–65. 10.1089/fpd.2015.1946. doi: 10.1089/fpd.2015.1946 . 26204443

[44] Rodrigues GL, Panzenhagen P, Ferrari RG, Paschoalin VMF, Conte-Junior CA. Antimicrobial resistance in nontyphoidal *Salmonella* isolates from human and swine sources in Brazil: a systematic review of the past three decades. Microbial drug resistance (Larchmont, N.Y.). 2020 Oct; 26(10):1260–70. 10.1089/mdr.2019.0475. doi: 10.1089/mdr.2019.0475 . 32412862

[45] Pan Y, Hu B, Bai X, Yang X, Cao L, Liu Q, et al. Antimicrobial resistance of non-O157 shiga toxin-producing *Escherichia coli* isolated from humans and domestic animals. Antibiotics (Basel). 2021 Jan; 10(1):74. 10.3390/antibiotics10010074. doi: 10.3390/antibiotics10010074 . 33466678 PMC7828786

[46] Khanam F, Rajib NH, Tonks S, Khalequzzaman M, Pollard AJ, Clemens JD, et al. Case report: *Salmonella enterica* serovar Paratyphi B infection in a febrile Ill child during enhanced passive surveillance in an urban slum in Mirpur, Dhaka. The American Journal of Tropical Medicine and Hygiene. 2020 Jul; 103(1):231–3. 10.4269/ajtmh.19-0958. doi: 10.4269/ajtmh.19-0958 . 32458786 PMC7356450

[47] Toboldt A, Tietze E, Helmuth R, Fruth A, Junker E, Malorny B. 2012. Human infections attributable to the D-tartrate-fermenting variant of *Salmonella enterica* serovar Paratyphi B in Germany originate in reptiles and, on rare occasions, poultry. Applied and Environmental Microbiology. 2012 Oct; 78(20):7347–57. 10.1128/AEM.01732-12. doi: 10.1128/AEM.01732-12 . 22885742 PMC3457084

[48] Hassan R, Tecle S, Adcock B, Kellis M, Weiss J, Saupe A, et al. Multistate outbreak of *Salmonella Paratyphi* B variant L(+) tartrate(+) and *Salmonella* Weltevreden infections linked to imported frozen raw tuna: USA, March-July 2015. Epidemiology and Infection. 2018 Aug; 146(11):1461–7. 10.1017/S0950268818001462. doi: 10.1017/S0950268818001462 . 29880080 PMC6063755

[49] Castellanos LR, van der Graaf-van Bloois L, Donado-Godoy P, Veldman K, Duarte F, Acuña MT, et al. Antimicrobial resistance in *Salmonella enterica* serovar Paratyphi B variant Java in poultry from Europe and Latin America. Emerging Infectious Diseases. 2020 Jun; 26(6):1164–73. https://wwwnc.cdc.gov/eid/article/26/6/19-1121_article. PMID: 32441616. 32441616 10.3201/eid2606.191121 PMC7258445

[50] Brandão MA, Lopes AT, Neta MT, de Oliveira RB, Rezende RP, Albuquerque GR, et al. Microbiological quality and prevalence of β-lactam antibiotic resistance genes in oysters (*Crassostrea rhizophorae*). Journal of Food Protection. 2017. 80:488–96. 10.4315/0362-028X.JFP-16-098. doi: 10.4315/0362-028X.JFP-16-098 . 28207310

[51] Ouchar Mahamat O, Kempf M, Lounnas M, Tidjani A, Hide M, Benavides JA, et al. Epidemiology and prevalence of extended-spectrum β-lactamase- and carbapenemase-producing *Enterobacteriaceae* in humans, animals and the environment in West and Central Africa. International Journal of Antimicrobial Agents. 2021 Jan; 57(1):106203. 10.1016/j.ijantimicag.2020.106203. 33075511

[52] Ben Said L, Jouini A, Alonso CA, Klibi N, Dziri R, Boudabous A, et al. Characteristics of extended-spectrum β-lactamase (ESBL)- and pAmpC beta-lactamase-producing *Enterobacteriaceae* of water samples in Tunisia. The Science of The Total Environment. 2016 Apr; 550:1103–9. 10.1016/j.scitotenv.2016.01.042. doi: 10.1016/j.scitotenv.2016.01.042 . 26871556

[53] Islam MS, Sobur MA, Rahman S, Ballah FM, Ievy S, Siddique MP, et al. Detection of *bla*_TEM_, *bla*_CTX-M_, *bla*_CMY_, and *bla*_SHV_ genes among extended-spectrum beta-lactamase-producing *Escherichia coli* isolated from migratory birds travelling to Bangladesh. Microbial Ecology. 2022 May; 83(4):942–50. 10.1007/s00248-021-01803-x. doi: 10.1007/s00248-021-01803-x . 34312710 PMC8313370

[54] Singh NS, Singhal N, Virdi JS. Genetic environment of *bla*_TEM-1_, *bla*_CTX-M-15_, *bla*_CMY-42_ and Characterization of integrons of *Escherichia coli* isolated from an Indian urban aquatic environment. Frontiers in Microbiology. 2018 Mar; 9:382. https://link.springer.com/article/10.1007/s00248-021-01803-x. doi: 10.3389/fmicb.2018.00382 . 29563901 PMC5845874

[55] Sellera FP, Fernandes MR, Moura Q, Carvalho MPN, Lincopan N. Extended-spectrum-β-lactamase (CTX-M)-producing *Escherichia coli* in wild fishes from a polluted area in the Atlantic Coast of South America. Marine Pollution Bulletin. 2018 Oct; 135:183–6. 10.1016/j.marpolbul.2018.07.012. doi: 10.1016/j.marpolbul.2018.07.012 . 30301029

[56] Cepas V, Soto SM. Relationship between virulence and resistance among gram-negative bacteria. Antibiotics (Basel). 2020 Oct; 9(10):719. 10.3390/antibiotics9100719. doi: 10.3390/antibiotics9100719 . 33092201 PMC7589547

[57] Martínez JL, Baquero F. Interactions among strategies associated with bacterial infection: pathogenicity, epidemicity, and antibiotic resistance. Clinical Microbiology Reviews. 2002 Oct; 15(4):647–79. 10.1128/CMR.15.4.647-679.2002. doi: 10.1128/CMR.15.4.647-679.2002 . 12364374 PMC126860

[58] Sandvig K. Shiga toxins. Toxinology. 2001 Nov; 39(11):1629–35. 10.1016/S0041-0101(01)00150-7. doi: 10.1016/S0041-0101 11595626

[59] Kim JS, Lee MS, Kim JH. Recent updates on outbreaks of shiga toxin-producing *Escherichia coli* and its potential reservoirs. Frontiers in Cellular and Infection Microbiology. 2020 Jun; 10:273. 10.3389/fcimb.2020.00273. doi: 10.3389/fcimb.2020.00273 . 32582571 PMC7287036

[60] Hitchcock PJ, Leive L, Mäkelä PH, Rietschel ET, Strittmatter W, Morrison DC. Lipopolysaccharide nomenclature-past, present, and future. Journal of Bacteriology. 1986 Jun; 166(3):699–705. 10.1128/jb.166.3.699-705.1986. doi: 10.1128/jb.166.3.699-705.1986 . 2872204 PMC215174

[61] van Asten AJ, van Dijk JE. Distribution of “classic” virulence factors among *Salmonella* spp. FEMS Immunology and Medical Microbiology. 2005 Jun; 44(3):251–9. 10.1016/j.femsim.2005.02.002. 15907446

[62] Sharma I, Das K. Detection of *invA* gene in isolated *Salmonella* from marketed poultry meat by PCR assay. Journal of Food Processing and Technology. 2016 Jan; 7:2. 10.4172/2157-7110.1000564.

[63] Kadry M, Nader SM, Dorgham SM, Kandil MM. Molecular diversity of the *invA* gene obtained from human and egg samples. Veterinary World. 2019 Jul; 12(7):1033–8. 10.14202/vetworld.2019.1033-1038. doi: 10.14202/vetworld.2019.1033-1038 . 31528029 PMC6702568

[64] Koonse B, Burkhardt W, Chirtel S, Hoskin GP. *Salmonella* and the sanitary quality of aquaculture shrimp. Journal of Food Protection. 2005 Dec; 68(12):2527–32. 10.4315/0362-028X-68.12.2527. doi: 10.4315/0362-028X-68.12.2527 . 16355822

[65] Patel A, Jeyasekaran G, Jeyashakila R, Anand T, Wilwet L, Pathak N, et al. Prevalence of antibiotic resistant *Salmonella* spp. strains in shrimp farm source waters of Nagapattinam region in South India. Marine Pollution Bulletin. 2020 Jun; 155:111171. 10.1016/j.marpolbul.2020.111171.PMID: doi: 10.1016/j.marpolbul.2020.111171.PMID: . 32469781

[66] Shabarinath S, Kumar HS, Khushiramani R, Karunasagar I, Karunasagar I. Detection and characterization of *Salmonella* associated with tropical seafood. International Journal of Food Microbiology. 2007 Mar; 114(2):227–33. 10.1016/j.ijfoodmicro.2006.09.012. doi: 10.1016/j.ijfoodmicro.2006.09.012 . 17141346

[67] Malorny B, Hoorfar J, Bunge C, Helmuth R. Multicenter validation of the analytical accuracy of *Salmonella* PCR: towards an international standard. Applied and Environmental Microbiology. 2003 Jan; 69(1):290–6. 10.1128/AEM.69.1.290-296.2003. doi: 10.1128/AEM.69.1.290-296.2003 . 12514007 PMC152403

[68] Saingam P, Li B, Yan T. Use of amplicon sequencing to improve sensitivity in PCR-based detection of microbial pathogen in environmental samples. Journal of Microbiological Methods. 2018 Jun; 149:73–9. 10.1016/j.mimet.2018.05.005. doi: 10.1016/j.mimet.2018.05.005 . 29746923

