# Supplementary material for: Antimicrobial resistance, Extended-Spectrum β-Lactamase production and virulence genes in Salmonella enterica and Escherichia coli isolates from estuarine environment
Source: PLoS One. 2023 Apr 28;18(4):e0283359. doi: 10.1371/journal.pone.0283359 (PMC10146452; doi:10.1371/journal.pone.0283359)
Supplement: Attachment — Submitted filename: Response to reviewers 190123.docx [file pone.0283359.s001.docx]

**PONE-D-22-28928**

Antimicrobial Resistance, Extended-Spectrum β-Lactamase Production and Virulence Genes in *Salmonella enterica* and *Escherichia coli* Isolates from Estuarine Environment

| **Comment** | **Response** |
| --- | --- |
| Reviewer #1: The study described a statistical model of the association between the most common AMR phenotype/ genotype, virulence genes among Salmonella and E. coli isolated from Bivalve mollusks, acting as an indicator for environmental faecal contamination and estuarine water. The study is interesting but the beta-lactamase sequences obtained should be submitted into the database and the accession numbers should be added in the manuscript without which the manuscript cannot be considered further. | The beta-lactamase sequences were deposited in NCBI and the accession number OQ282894-OQ282896 was added to the manuscript in lines 130-133 as follows: “The result of the DNA sequence was blasted and aligned with reference embedded in GenBank database available from the National Centre for Biotechnology Information (NCBI) (http://www.ncbi.nlm.nih.gov/ BLAST) (accession number OQ282894-OQ282896)”. |
| Line 38. Please replace the old reference with current global human mortality rate attributed to AMR | The number of infections and deaths due to antimicrobial resistance was updated and the new reference was replaced.  Reference  CDC. Antibiotic resistance. Centers for Disease Control and Prevention, National Center for Emerging and Zoonotic Infectious Diseases (NCEZID), Division of Healthcare Quality Promotion (DHQP). https://www.cdc.gov/drugresistance/about.html. 2022. |
| Line 86. ‘not exposed to antimicrobials’ | The error has been corrected as suggested. |
| Line 90. Did you conduct any qualitative survey to know the kind of antibiotics used in human and veterinary medicine? | We did not conduct a survey on antimicrobial use, but only addressed the list of antimicrobials used in this study. However, the sentence in lines 90-91 was deleted. |
| Line 134. ‘invasion’ should be replaced with ‘invasin’ | The word “invasion” was replaced as suggested. |
| Line 135-138. PCR conditions, primer sequences or reference should be added. | PCR conditions, primer sequences, and reference were already added in Tables 1-3. |
| Line 183. cannot be considered as ESBL | BlaTEM was removed because it was not considered an ESBL positive isolate. |
| Line 185. CTX-M types should be mentioned obtained in nucleotide sequencing | The CTX-M nucleotide sequence was added in lines 187-188 as follow “Eight (2.0%) of 409 *E. coli* isolates from estuarine water were ESBL producers, of which three isolates were positive for *bla*_CTX-M-55_.” |
| Line 231. Did the local people use estuarine water for drinking/domestic purpose? | In this study site, locals use estuarine water only for aquaculture. Therefore, lines 234-237 were modified as “Humans can be infected with AMR bacteria by eating aquatic animals or direct contact with contaminated environment”. |
| Line 239. How did the authors know that sulfamethoxazole is commonly used in human and veterinary medicine in local settings? | The sentence “The high prevalence of sulfamethoxazole observed in this study may be widely used in human and animal medicine because this antimicrobial can be used to treat and prevent many bacterial infections at affordable cost [31].” and reference is added for clarification in lines 243-244.  Reference  31. Kemnic TR, Coleman M. Trimethoprim Sulfamethoxazole. In: StatPearls. Treasure Island (FL): StatPearls Publishing; 2022 Jan; https://www.ncbi.nlm.nih.gov/books/NBK513232/. |
