# Supplementary material for: Antimicrobial resistance, Extended-Spectrum β-Lactamase production and virulence genes in Salmonella enterica and Escherichia coli isolates from estuarine environment
Source: PLoS One. 2023 Apr 28;18(4):e0283359. doi: 10.1371/journal.pone.0283359 (PMC10146452; doi:10.1371/journal.pone.0283359)
Supplement: Attachment — Submitted filename: A rebuttal letter PlosOne 060323.docx [file pone.0283359.s002.docx]

**A rebuttal letter**

The reviewer has noticed that the accession numbers cited in pages 130-133 are not found in GenBank NCBI database. Please give the correct accession numbers that can be accessed by the readers.

The accession numbers cited on pages 130-133 have been published in GenBank database (http://www.ncbi.nlm.nih.gov/ BLAST) (accession number OQ282894-OQ282896).
